# Functional dental status and oral health-related quality of life in an over 40 years old Chinese population

**DOI:** 10.1007/s00784-012-0834-x

**Published:** 2012-09-27

**Authors:** Qian Zhang, Dick J. Witter, Anneloes E. Gerritsen, Ewald M. Bronkhorst, Nico H. J. Creugers

**Affiliations:** 1 Department of Prosthetic Dentistry, Affiliated Hospital of Medical School, Qingdao University, Jiangsu Road 16#, Qingdao, People’s Republic of China; 2 Department of Oral Function and Prosthetic Dentistry, College of Dental Science, Radboud University Nijmegen Medical Centre, Philips van Leydenlaan 25, 6525 EX Nijmegen, The Netherlands; 3 Department of Preventive and Restorative Dentistry, College of Dental Science, Radboud University Nijmegen Medical Centre, Philips van Leydenlaan 25, 6525 EX Nijmegen, The Netherlands

**Keywords:** Oral health-related quality of life, Occlusal status, Hierarchical dental functional classification system, Chinese adults

## Abstract

**Objectives:**

This study aimed to assess oral health-related quality of life (OHRQoL) related to dental status.

**Material and methods:**

One thousand four hundred sixty-two Chinese subjects over 40 years, dentate in both jaws, were categorized in a hierarchical functional classification system with and without tooth replacements. OHIP-14CN scores were used to assess OHRQoL and analyzed using multivariable logistic regression including five dental conditions (‘≥10 teeth in each jaw’; ‘complete anterior regions’; ‘sufficient premolar regions’ (≥3 posterior occluding pairs (POPs)); ‘sufficient molar regions’ (bilaterally ≥1 POP); and tooth replacement) after adjustment for five background variables. Likelihood ratios for impaired OHRQoL (OHIP total score ≥5) were assessed at each level of the classification system.

**Results:**

In the hierarchical scheme, OHIP-14CN total scores were highest in branch ‘<10 teeth in each jaw’ (8.5 ± 9.5 to 12.3 ± 13.2). In branch ‘≥10 teeth’ scores ranged from 6.2 ± 7.7 to 8.3 ± 9.3. The most important dental condition discriminating for impact on OHRQoL was ‘≥10 teeth in each jaw’ (Likelihood ratio 1.59). In this branch subsequent levels were discriminative for impaired OHRQoL (Likelihoods 1.29–1.69), in the branch ‘<10 teeth in each jaw’ they were not (Likelihoods 0.99–1.04). Tooth replacements were perceived poorer as their natural counterparts (odd ratios, 1.30 for fixed and 1.47 for removable appliances).

**Conclusions:**

OHRQoL was strongly associated with the presence of at least 10 teeth in each jaw. The hierarchical classification system predicted approximately 60 % of subjects correctly with respect to impaired OHRQoL.

**Clinical relevance:**

From an OHRQoL perspective, natural teeth were preferred over artificial teeth.

## Introduction

Tooth loss is associated with adverse oral health-related quality of life (OHRQoL). Besides number, location and distribution of missing teeth also affect the severity of impairment [1]. The results of a systematic review suggest that the number of occluding pairs of teeth is an important predictor of OHRQoL, and that the prevalence of adverse OHRQoL increases sharply once the number of teeth present drops below 20 [1]. The associations found in the systematic review seemed to be independent from the OHRQoL instrument used as well as from the context of the included samples. However, it has also been stated that the extent and severity of impairment probably are context dependent [2]. Although associated, the correlation between the number of missing teeth and the number of occluding pairs (which is a derivative of the distribution of missing teeth) is not linear [3]. As a result, it was concluded that the role of cultural background, as well as location and distribution of missing teeth in perceived OHRQoL remains subject for further exploration [1].

Tooth loss above the age of 40 years is a common phenomenon in China [4, 5]. The number of missing teeth in Chinese adults increases with age from approximately two at the age of 40 years to approximately 12 at the age of 65 years [4]. One of the most immediate and important functional consequences of tooth loss is a reduction in chewing ability. Recent studies have indicated that impaired chewing ability affects OHRQoL [6, 7]. As oral rehabilitation restores partially the objective chewing capacity it may improve OHRQoL. It has been reported that the correlation between perceived chewing ability and OHRQoL is not substantially influenced by number of teeth and age, but by gender, level of education, treatment demand, and prosthodontic status [6, 8]. In a Swedish study among subjects 50–70 years of age that assessed the relationship between OHRQoL and dental and prosthodontics status, the number of remaining teeth appeared to have more impact than the type of denture used to replace missing teeth [9]. In this population removable dental prostheses (RDP) did not constitute an important factor in determining OHRQoL. However, in a Danish patient population it appeared that RDP treatment improved OHRQoL, but caused new problems [10]. In that study, fixed dental prostheses (FDP) treatment also improved OHRQoL, but less than RDP treatment. A study in a Finnish population indicated that among participants with 20 or more teeth, those wearing RDP were more likely to report oral impacts than those who did not. In contrast, in subjects with fewer than 20 teeth RDP replacement was associated with better OHRQoL [11].

It has been pointed out that perceptions of oral impacts in relation to dental status might differ between Western and Eastern populations [12]. For example, in a 60–80 years old Chinese population, partially dentate subjects experienced adverse impact on OHRQoL from RDP [13]. A study among Chinese subjects aged 55 years and older showed weak but significant associations between number of teeth, occluding pairs, and impacts on OHRQoL [12]. It should be noted that these associations were stronger when replaced teeth and replaced occluding pairs of teeth were taken into account. Interestingly, Japanese studies investigating denture quality of RDP reported poorer OHRQoL for subjects with ‘bad’ RDPs compared to subjects with ‘good’ RDPs, but minimal impacts on health-related quality of life as mediated by OHRQoL [14, 15].

**Fig. 1:**
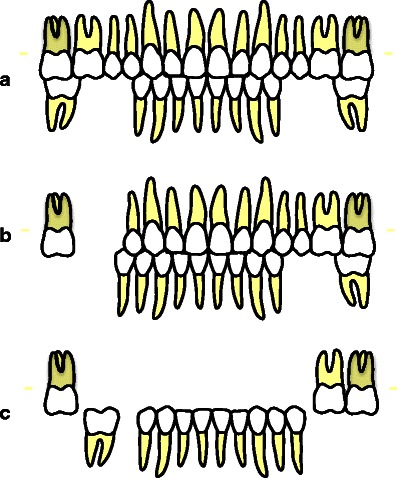
Examples of reduced dentitions categorized according to the Eichner index: **a** Eichner class A2 (posterior occluding pairs in four supporting zones); **b** Eichner class B1 (posterior occluding pairs in three supporting zones); **c** Eichner class C1 (no antagonistic contacts)

The majority of studies addressing the relationship between dental status and OHRQoL, used the number of teeth and occluding pairs as a measure for dental status. However, the actual configurations of the dentitions involved remain unclear. Recently, the widely used Eichner index, which classifies posterior occlusal support by occlusal contacts between maxillary and mandibular premolar and molar zones, has been validated for an elderly Japanese population with respect to chewing ability [16]. The Eichner index characterizes dentitions according to the presence of occlusal supporting zones (Fig. 1). Eichner class A includes dentitions with four occlusal supporting zones, which means that at least one posterior occluding pair in both premolar and molar area at each side is present. Eichner class B includes dentitions with three (B1), two (B2), or one (B3) supporting zone, or support in the anterior area only (B4). Eichner class C includes dentitions without antagonistic occluding contacts. The Eichner index has been considered a feasible modulating factor in oral wellbeing with regard to prosthodontics status [17]. However, the Eichner index does not specify the exact number and location of teeth and is not reflecting oral functionality in detail.

Recently a hierarchical dental functional classification system has been introduced that reflects oral functionality [3, 18–20]. This classification system is based on the conclusions of a systematic review that sufficient oral function depends on the presence of minimally 20 teeth with nine to 10 occluding pairs, no tooth loss in the anterior region, and the retention of premolars, whereas there may be little increase in satisfaction with teeth seen in subjects with presence of molars [21]. In the functional classification system oral functionality is expressed by combining (1) number of teeth in upper and lower jaw, (2) completeness of anterior regions, (3) number of premolar occluding pairs, and (4) number of molar occluding pairs.

Recently, we reported analyses of data from a large epidemiological study in a population over 40 years in Shandong, China, using the hierarchical dental functional classification system as described above. One report was addressing tooth loss and tooth replacements and dental functionality; another addressed chewing ability. The aim of the present study was to analyze the relationship between dental and prosthodontic status and OHRQoL. It was hypothesized that subjects with less than 10 teeth in each jaw, incomplete anterior regions, and impaired (pre)molar regions had poorer OHRQoL compared to their counterparts. Furthermore, it was hypothesized that artificial teeth, either fixed or removable, do not compensate for missing natural teeth.

## Materials and methods

The study was conducted in 2009 and 2010 in the Qingdao area, located at the east coast of Shandong Province, situated in Eastern China. Shandong is one of the largest provinces in China in terms of population (94 million in 2008) and economy. Qingdao area has approximately 8 million inhabitants, of which 3 million live in Qingdao city and 5 million in the surrounding rural territory. The area includes five county-level cities (approximately 200.000 to 400.000 residents each) and surrounding counties. Each rural county comprises 40–80 small rural villages. The total area of Qingdao (urban and rural) is approximately 10,000 km^2^ with a coastline of approximately 730 km.

### Sampling method

Details about the sampling method have been published in previous papers [4, 19]; a short summary is presented here. For this study, a cross-sectional survey, representing 1,588 subjects aged ≥40 years living in urban and rural areas in Qingdao, Shandong Province, was conducted. Subjects were selected randomly from administrative lists of residents of communities or villages provided by local authorities and lists of employees of factories. Inclusion aimed at proportional distribution according to age categories, gender, and place of residence (urban or rural).

The urban sample comprised 11 communities and four factories in Qingdao City. Subjects of certain age categories were underrepresented in the initial urban sample. As truly representative sampling was not feasible, the pathfinder sampling method was adopted to cover relevant groups of the population intended [22]. For the rural sample, one representative county (Zhugou) was chosen. This county—predominantly agrarian—comprises 56 villages ranging from 153 to 1,583 inhabitants. As gross domestic product (GDP) is related with socio-economic status (SES), 10 villages with different GDP were selected randomly.

The research was carried out in compliance with the Helsinki Declaration and was approved by the ethics committee of the medical school at Qingdao University, Qingdao, China.

### Participants

Of the 1,588 subjects participating in the epidemiological study, 126 subjects (8 %) were edentulous in one or both jaws and were excluded from the present analyses. The remaining 1,462 subjects dentate in both upper and lower jaw were included (Table 1). A previous report of this survey revealed that of all dentate subjects, 59 % (*n* = 861) had a natural dentition without any tooth replacement, 30 % had FDPs (*n* = 441), and 11 % (*n* = 160) had RDPs. Forty-three subjects (3 % of the total sample) had both FDP and RDP. The majority of subjects with FDP (57 %) had one or two teeth replaced; the majority of RDPs (78 %) replaced three or more teeth [19]. More details with respect to number of teeth and tooth replacements have been described in that report.

**Table 1:** Number (%) of included subjects (*n* = 1,462) dentate in upper and lower jaw according to gender and place of residence, distribution of SES, and age (minimum, maximum, and mean)

	Urban	Rural	Total
Female	405 (58)	297 (42)	702 (48)
Male	385 (51)	375 (49)	760 (52)
Total	790 (54)	672 (46)	1,462 (100)
	SES high	SES middle	SES low
SES^a^	583	449	428
	Minimum	Maximum	Mean (SD)
Age	40	87	54.9 (10.5)

^a^SES data of two subjects missing

### Questionnaire

Subjects were asked to complete a structured questionnaire that was used previously in a study in Vietnam [3] and translated into Mandarin. This Chinese version was checked for language adequacy by a panel of dentists and pilot tested on 20 Chinese subjects to assess clarity. Some minor modifications were made based on the results of the pilot. The questionnaire included demographic information (age, gender, place of residence (urban, rural)) and SES. For assessment of SES (high, middle, and low) a modified Kuppuswamy classification was used [23], which is based on the subject’s level of education (five levels: higher education; college; primary school; no formal education, literate; no formal education, illiterate), occupation (three levels: white collar = office worker, teacher, doctor and academic researcher, and government officers; service people = salespeople, house worker and vehicle driver; blue collar = farmer, factory worker, forestry worker, fisher, and (lower) military personal), and household income (four levels: income covers expenses, no loans needed; income does not cover expenses, no loans needed; income covers expenses, loans needed incidentally; and income does not cover expenses’, loans needed regularly).

The Chinese short version of the Oral Health Impact Profile (OHIP-14CN) was included to assess the psychosocial impact of oral disorders on quality of life. The Chinese version of OHIP-14 has been validated for implementation in OHRQoL research [24]. Responses on each OHIP question were given on a five-point Likert scale (0 = never; 1 = hardly ever; 2 = occasionally; 3 = fairly often; 4 = very often) for a reference period of 3 months.

It appeared that all subjects understood Mandarin. However, subjects not able to complete the questionnaire themselves (e.g., because of illiteracy or visual impairment) were helped by an assistant who read aloud the questions and recorded the answers. After completion, the questionnaire was checked for unrecorded items, and if applicable, subjects were requested to complete the form.

### Clinical examination

After obtaining verbal consent from the participants, a clinical examination was conducted by a calibrated examiner following the procedures and diagnostic criteria recommended by the World Health Organization [25]. Inter-observer agreements between the principal investigator and experienced researchers in the field on DMFT variables were excellent (kappa’s ≥0.89). Of all variables recorded, only the presence of teeth (including third molars), tooth type, number, and location of posterior occluding pair of natural teeth (POPs) and tooth replacements were considered in the present study. Roots were considered as missing teeth. A distinction was made between teeth replaced by FDP and those replaced by RDP. A replaced tooth was defined as a missing tooth replaced by either FDP or RDP. Mean numbers of POPs added by FDP or RDP were also considered.

### Dental functional status classification system

Subjects were classified on the basis of a dichotomized five level branching hierarchy of dental functional status in which the criteria applied on the levels are based on conditions that reflect functionality [20, 21] (Table 2). With regard to each level in the branching hierarchy, the number of natural teeth, the tooth types present, and the number of natural POPs were calculated. Subjects were classified in two ways. First, subjects were classified on the basis of their configuration of natural teeth only (Class_nat_). Next they were reclassified on the basis of configurations including natural teeth plus teeth replaced by FDP (Class_F_) or by RDP (Class_R_).

**Table 2:** Levels and criteria for dichotomization of the step-by-step branching hierarchy used in the subsequent categories

Level	Meeting criterion		Dichotomy
	Yes	No	
I Dentition level	≥1 Tooth present in each jaw		≥1 Tooth vs. no teeth
II Jaw level	≥10 Teeth in both upper *and* lower jaw	<10 Teeth in upper *or* lower jaw	≥10 Teeth vs. <10 teeth
III Anterior level	All 12 anterior teeth present	<12 Anterior teeth	Complete vs. incomplete
IV Premolar level	3 or 4 Occluding pairs of premolars	≤2 Occluding pairs of premolars	‘Sufficient’ vs. ‘impaired’
V Molar level	≥1 Occluding pairs of molars at both left *and* right side of the dentition	No occluding pairs of molars at left *or* right side of the dentition	‘Sufficient’ vs. ‘impaired’

### Data analyses

Two approaches were used to analyze OHIP-14CN in relation to dental conditions. First, the relationships were analyzed for the conditions of the different dental regions simultaneously. In the second approach, oral health-related quality of life was related to the hierarchical functional classification system, in which the dental regions are considered sequentially.

In the first approach—in which the relationship between OHIP-14CN and the separate dental regions is analyzed—multivariable logistic regression models were used. In these models OHIP-14CN total scores were dichotomized using the sample median score as the cut-off point: total scores less than 5 (the sample median score) were considered as ‘not impaired OHRQoL’; total scores ≥5, were considered as ‘impaired OHRQoL’. The dichotomized OHIP-14CN total score was the dependent variable; the conditions at the levels II to V (≥10 teeth in each jaw; anterior region complete; premolar region sufficient; and molar region sufficient), and tooth replacement were the independent variables. Possible associations between the condition of the separate dental regions and OHIP-14CN total score were adjusted for five background variables: questionnaire administration (completely self-administered vs. (partly) assisted by a dental assistant); the demographic variables age (age categories 40–49, 50–59, 60–69 years, and 70 years and older), gender, and place of residence (urban or rural); and SES (three levels).

The performance of the multivariable logistic models was expressed as the percentages of subjects having impaired OHRQoL predicted correctly by (1) the dental conditions only, and (2) all 10 variables. To express the performance of the logistic models the AUC statistic (area under the curve) is used. An AUC of 0.5 indicates a total absence of model fit; an AUC of 1 belongs to a situation where model fit is perfect. Although the models are etiologic by nature and not meant as a predictive tool, the percentage predicted correctly are presented as an additional indication of the model fit.

In the second approach—in which the relationship between OHRQoL and dental functional status is analyzed, Likelihood ratios were calculated after dichotomization (meeting vs. not meeting the respective dental conditions). These Likelihood ratios express the extent to which a given condition, for instance having at least 10 teeth in each jaw, discriminates between people with and without OHRQoL impacts. A Likelihood ratio of 1 indicates a classification criterion that is not discriminatory. Both approaches were applied to the dental conditions based on (1) natural teeth only (Class_nat_) and subsequently (2) on levels based on natural teeth plus teeth replaced by FDP (Class_F_) and by RDP (Class_R_).

R software version 2.13.1 was used for the statistical analyses [26].

## Results

The distribution of subjects categorized according to the condition of meeting/not meeting a functional level in the hierarchical classification system is shown in Table 3. On the basis of considering natural teeth only (Class_nat_), 766 (52 %) subjects met all criteria for a functional dentition, while 159 (11 %) subjects met none of the criteria. On the basis of considering natural teeth plus teeth replaced by FDP (Class_F_), these numbers increased to 896 (61 %), respectively decreased to 132 (9 %); considering natural teeth plus teeth replaced by RDP (Class_R_) the numbers were 836 (57 %) respectively 96 (7 %).

**Table 3:** Distribution of subjects (*n* = 1,462) according to the condition of meeting/not meeting a functional level in the hierarchical classification system based on natural teeth only (Class_nat_), natural teeth plus teeth replaced by FDP (Class_F_), and natural teeth plus teeth replaced by RDP (Class_R_)

Condition			Molar region ‘sufficient’								
			Class_nat_			Class_F_			Class_R_		
			Yes	No	Total	Yes	No	Total	Yes	No	Total
>10 teeth in each jaw	Anterior region complete	Premolar region ‘sufficient’									
Yes	Yes	Yes	766	114	880	896	100	996	836	119	955
		No	73	40	113	58	31	89	75	38	113
	No	Yes	148	32	180	130	24	154	156	29	185
		No	29	3	32	16	4	20	26	3	29
No	Yes	Yes	0	6	6	0	2	2	0	7	7
		No	8	39	47	7	34	41	6	31	37
	No	Yes	3	20	23	2	12	14	4	17	21
		No	22	159	181	14	132	146	19	96	115
Total					1,462			1,462			1,462

### OHRQoL and dental conditions determined by natural teeth only

For different dental conditions odd ratios for having impaired OHRQoL ranged from 0.72 to 1.05, however, were not statistically significant (Table 4). The variable ‘tooth replacement’ also did not influence the chance for having impaired OHRQoL significantly (OR = 1.18; *p* = 0.20), meaning that subjects with tooth replacement did not differ from subjects without tooth replacements with respect to impact of dental conditions on OHRQoL. In the model based on the dental conditions only, the percentage of correctly predicted subjects with impaired OHRQoL was 57.6; the AUC was 0.584, showing a moderate level of predictability of the model. The full model, including all 10 variables, predicted 59.2 % while the AUC was 0.613.

**Table 4:** Odds ratios [95 % CI] for having impaired OHRQoL according to the dental conditions in the multivariable logistic regression model, adjusted for the background variables age, gender, place of residence, SES, and questionnaire administration format

Condition^a^ (level)	OR	*p* value	95 % CI
≥10 teeth in each jaw (II)	1.05	0.84	[0.67…1.64]
Anterior regions complete (III)	0.80	0.14	[0.60…1.08]
Premolar region ‘sufficient’ (IV)	0.72	0.06	[0.52…1.01]
Molar region ‘sufficient’ (V)	0.76	0.08	[0.56…1.03]
Tooth replacement	1.18	0.20	[0.92…1.50]
AUC dental conditions	0.584		
Percentage correctly predicted by dental conditions only	57.6		
AUC dental conditions plus background variables	0.613		
Percentage correctly predicted by dental conditions plus background variables	59.2		

Conditions based on configuration of natural teeth only (Class_nat_)

*AUC* area under curve

^a^Reference = condition not present

**Fig. 2:**
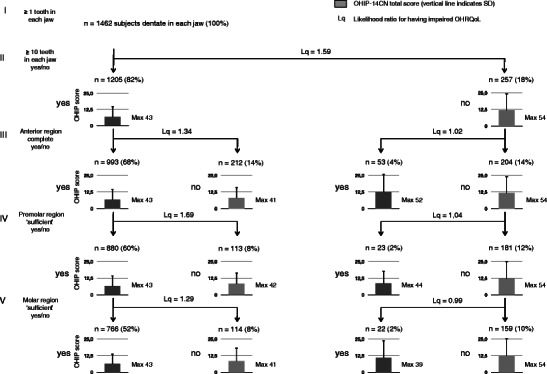
Distribution of subjects dentate in each jaw (*n* = 1,462) according to the functional classification system based on natural teeth only [3], OHIP-14CN total scores (*vertical bars* indicate SD) and Likelihood ratios for impaired OHRQoL: *I* dentate in each jaw, *II* ≥10 natural teeth in each jaw, *III* anterior region complete, *IV* premolar region ‘sufficient’, *V* molar region ‘sufficient’. *Dark columns* indicate status of meeting the criterion

The branching hierarchy shown in Fig. 2 describes 82 % of all subjects dentate in each jaw (*n* = 1,462) up to level IV (premolar region) and 72 % up to level V (molar region). In Fig. 2, categories of subjects not meeting the dental conditions in the ‘≥10 teeth in each jaw’ branch and categories meeting the dental conditions in the ‘<10 teeth in each jaw’ branch were not further dichotomized to the next level. OHIP-14CN total scores were highest in the branch ‘<10 teeth in each jaw: they ranged from 8.5 (SD 9.5) for subjects with an incomplete anterior region and ‘sufficient’ premolar region to 12.3 (SD 13.2) for subjects that met none of the criteria. In the ‘≥10 teeth in each jaw’ branch, OHIP-14CN total scores ranged from 6.2 (SD 7.7) to 8.3 (SD 9.3) being lowest for subjects that met all criteria and highest for subjects with complete anterior region, ‘sufficient’ premolar region but ‘impaired’ molar region. The Likelihood ratio for having impaired OHRQoL at level II of the classification system (‘≥10 teeth in each jaw’) was 1.59 (i.e., if the criterion was not met). Highest Likelihood ratio in the ‘≥10 teeth in each jaw’ branch was found for the premolar region (level IV: L = 1.69). In the branch of ‘<10 teeth in each jaw’, Likelihood ratios for having impaired OHRQoL ranged from 0.99 to 1.04. This means that if subjects had less than 10 teeth in each jaw discriminative potential from meeting vs. not meeting the criteria at the different functional levels was lacking.

### OHIP-14CN and dental conditions based on natural teeth plus teeth replaced by FDP or RDP

After reclassification to categories based on natural teeth plus tooth replacements (Class_F_ and Class_R_) subjects with FDP more often reported impaired OHRQoL than subjects of the same classification with natural teeth only: OR = 1.30 (*p* = 0.03; Table 5). The same was seen for subjects with RDP compared to their counterparts with natural teeth only: OR = 1.47 (*p* = 0.03). In Class_F_, the conditions ‘anterior region complete’ and ‘sufficient premolar region’ were associated significantly with less impaired OHRQoL (ORs respectively 0.73 (*p* = 0.04) and 0.63 (*p* = 0.02)), whereas in Class_R_ subjects with ‘sufficient’ molar regions reported significantly less often impaired OHRQoL (OR = 0.68; *p* = 0.01). On the basis of the dental conditions only, the percentages of correctly predicted subjects with impaired OHRQoL were 56.2 (AUC = 0.567) for Class_F_ and 57.1 (AUC = 0.570) for Class_R_. The full model predicted respectively 59.0 and 59.7 % (AUCs 0.610 and 0.618) of subject with impaired OHRQoL correctly.

**Table 5:** Odds ratios, *p* values, and 95 % confidence intervals (CI) of the multivariable logistic regression analysis for having impaired OHRQoL with dental status after reclassification to Class_F_ and Class_R_, adjusted for the background variables age, gender, place of residence, SES, and questionnaire administration format

Condition^a^ (level)	In Class_F_			In Class_R_		
	OR	*P*	95 % CI	OR	*P*	95 % CI
≥10 teeth in each jaw (II)	1.05	0.85	0.63–1.76	0.92	0.74	0.57–1.48
Anterior regions complete (III)	**0.73**	0.04	0.54–0.99	0.79	0.11	0.60–1.05
Premolar region ‘sufficient’ (IV)	**0.63**	0.02	0.43–0.92	0.81	0.23	0.58–1.14
Molar region ‘sufficient’ (V)	0.83	0.27	0.60–1.15	**0.68**	0.01	0.50–0.92
Tooth replacement	**1.30**	0.03	1.02–1.65	**1.47**	0.03	1.04–2.08
AUC dental conditions	0.567			0.570		
Percentage subjects correctly predicted by dental conditions only	56.2			57.1		
AUC dental conditions plus background variables	0.610			0.618		
Percentage subjects correctly predicted by dental conditions plus background variable	59.0			59.7		

Bold figures indicate significant relationships

*AUC* area under curve

^a^Reference = condition not present

In general, Likelihood ratios for having impaired OHRQoL for subjects classified in the hierarchical scheme on the basis of natural teeth plus replaced teeth (Class_F_/Class_R_) were higher than Likelihoods for subjects classified on the basis of natural teeth only (Class_nat_) except for premolar region ‘impaired’ (in both Class_F_ and Class_R_) and molar region ‘impaired’ (in Class_F_; Table 6). Likelihood ratios for having impaired OHRQoL were highest at the level of ‘10 teeth in each jaw’ yes/no (1.70 in Class_F_ and 1.71 in Class_R_). For the subsequent predictors, Likelihood ratios for having impaired OHRQoL were generally higher if the conditions at proceeding levels were met than if the conditions at preceding levels were not met. For instance, if the subjects had at least 10 teeth in each jaw and a complete anterior region, the predictor ‘premolar region impaired’ revealed Likelihood ratios for impaired OHRQoL of 1.64 in Class_F_ and 1.57 in Class_R_. If subjects did not meet these conditions, Likelihood ratios were respectively 1.09 and 1.00.

**Table 6:** Likelihood ratios for having impaired oral health-related quality of life according to the condition of meeting/not meeting a functional level in the hierarchical classification system, based on natural teeth only (Class_nat_) and on natural teeth plus replaced teeth (Class_F_/Class_R_)

Condition			Predictor			
≥10 teeth in each jaw	Anterior region complete	Premolar region ‘sufficient’		Class_nat_	Class_F_	Class_R_
			<10 teeth in each jaw	1.59 (103)	1.70 (78)	1.71 (69)
Yes			Anterior region incomplete	1.34 (99)	1.42 (78)	1.28 (101)
No			Anterior region incomplete	1.02 (22)	1.04 (18)	1.00 (17)
Yes	Yes		Premolar region ‘impaired’	1.69 (48)	1.64 (38)	1.57 (49)
No	No		Premolar region ‘impaired’	1.04 (11)	1.09 (16)	1.00 (8)
Yes	Yes	Yes	Molar region ‘impaired’	1.29 (56)	1.09 (46)	1.33 (58)
No	No	No	Molar region ‘impaired’	0.99 (8)	0.97 (4)	1.03 (8)

Numbers in parenthesis reflect the smallest number of subjects in the four cells in the respective comparisons

## Discussion

This study aimed to investigate OHRQoL in terms of OHIP-14CN total scores in Chinese adults over 40 years without and with prosthodontic replacements. As a result of the sampling method (described in detail in Zhang et al. [4]), the sample is considered to reflect the population in Shandong Province. However, in the present study subjects with one or both jaws edentulous (8 %) were not considered.

For the cut-off for OHRQoL impairment, the median OHIP-14CN total score was chosen, which was in this study 5 units. This score can be considered rather ‘stringent’ compared to other studies. For Australians aged 45–65 years OHIP-14 severity scores ranged from 5 to 12 units [27], partially dentate Japanese (mean age 63 ± 11.5 years) reported mean OHIP-14 scores of 13 ± 9 units [6], and for Swedish subjects the mean severity scores dropped from 7.5 units at the age of 40 years to 6 units for 70-year olds [28]. Hong Kong Chinese adults with orofacial pain symptoms had a mean total score of 10.1 (SD 9.4) [29].

The findings of this study with respect to tooth replacements confirm recent outcomes from a study among dentate subjects of 55 years and older in Southern China in which it was found that oral impacts were more strongly associated with clinical indicators based on natural teeth plus replaced teeth compared to clinical indicators based on natural teeth only [12]. However, the outcomes of the present study present differentiation between fixed and removable replacement teeth as well as among dental regions. The separate dental conditions in the different dental regions based on natural teeth only (Class_nat_), were not crucial for perceiving OHRQoL impacts, at least not significantly (Table 4). Also the variable ‘tooth replacement’ showed no significant associating with OHRQoL. For each level subjects with FDP or RDP replacements had similar OHRQoL impacts compared to their counterparts with similar dental conditions without these replacements. This indicates that the group of subjects with tooth replacement cannot be considered as ‘complainers’ if compared to their counterparts. In Class_F_, which is based on natural teeth plus fixed replacements, anterior regions that were ‘completed’ by FDP and premolar regions that were ‘altered’ to ‘sufficient’ by FDP were significantly associated with less chance for having impaired OHRQoL (Table 5). This means that fixed tooth replacements in these regions seem to improve OHRQoL. FDP replacement in the molar region was not associated with improved OHRQoL in this population. In contrast, in Class_R_ RDP replacements improved OHRQoL by ‘altering’ molar regions from ‘impaired’ to ‘sufficient’, but not by ‘completing’ the anterior region or ‘restoring’ the premolar region to the level of being ‘sufficient’. When analyzing associations between the variable ‘having tooth replacements or not’ with OHRQoL, subjects with FDP and RDP had significantly higher odds for impaired OHRQoL than their counterparts with similar dental conditions without these replacements (Table 5). Referring to the hypothesis, apparently the presence of FDP and RDP did improve OHQRoL by ‘restoring’ the separate dental regions, but these appliances themselves apparently were associated with negative side effects. From an OHRQoL perspective subjects seem to prefer natural teeth above artificial teeth. In this respect, the quality of the tooth replacements, including the indications for FDP and RDP, might play a role. With respect to FDPs, especially in rural areas in China, dental care providers (which are not necessarily dentists) prefer to apply rather unconventional prosthodontic principles, in which they tend to provide FDPs for low prices rather than RDPs, even when only very few teeth are available as abutment teeth [30].

In the hierarchical dental functional classification system, which describes the functional indicators combined, the most important dental condition that discriminates for impact on OHRQoL is the presence of at least 10 teeth in each jaw. Subjects with less than 10 teeth in each jaw generally reported more often (Likelihood ratio 1.59) and more severe impairment of OHRQoL (mean OHIP total score 11.8 units) compared to subjects with at least 10 teeth in each jaw (mean OHIP total score 6.8 units). This difference may be considered as just clinically relevant when using the threshold proposed by Allen et al. as a reference [31]. In that study the minimally important difference was calculated to be 9 units using an OHIP-20 questionnaire. On the other hand, the minimally important difference for the OHIP-49 questionnaire for prosthodontics treatment outcomes was found to be 6 OHIP units (95 % confidence interval, 2–9) [32]. Consequently, the hypothesis that having less than 10 teeth in each jaw is associated with clinically relevant OHRQoL impairment can be confirmed.

In the hierarchical classification the Likelihood ratios for impaired OHRQoL depends on the ‘branch’. In the ‘good’ branch (having ‘≥10 teeth in each jaw’, Likelihoods for impaired OHRQoL discriminate with respect to meeting or not meeting the dental conditions at the subsequent levels (Likelihoods from 1.29 to 1.69). In the ‘poor’ branch (having ‘<10 teeth in each branch’, Likelihoods are approximately 1 (0.99–1.04). A possible explanation might be that the dental configurations with less than 10 teeth in each branch is that deteriorated that further ‘detailed’ descriptions at the subsequent levels are meaningless for further discrimination with respect to OHRQoL, regardless whether subjects were classified based on natural teeth only (Class_nat_), or classified based on natural teeth plus FDPs (Class_F_) or plus RPDs (Class_R_) (Table 6). The hypothesis that incomplete anterior region and impaired (pre)molar region result in poorer OHQRoL can be confirmed only for the ‘good’ branch.

In the present study, the dental conditions predicted approximately 57 % of subjects correctly with respect to impaired OHRQoL. When the full model including background variables was applied this percentage raised marginally to 59. This suggests that the dichotomies in the hierarchical classification scheme are moderate indicators for OHRQoL impacts, whereas the included background variables only showed a marginal explanatory value. Limitations in associations between dental indicators and OHRQoL have been found in several studies [6, 11, 33–37]. As in Western studies, expectations and preferences regarding tooth replacements as well as psychological and social values most probably also influence impacts on OHRQoL in China.

The hierarchical dental functional classification system applied in the present study has been previously evaluated [3, 18–20]. The homogeneities of the dichotomized groups with respect to number of teeth and posterior occluding pairs were satisfactory [3, 19], while the functional dental status in the groups predicted chewing ability fairly strong [18, 20]. The findings of the present study contribute to the feasibility of the hierarchical functional dental classification system and add to the validity demonstrated in the previous studies.

## Conclusions

In this Chinese adult population:

- The most important dental condition in the hierarchical dental functional classification system that discriminates for impact on OHRQoL was the presence of at least 10 teeth in each jaw.
- The dental conditions of the hierarchical dental functional classification system predicted approximately 60 % of subjects correctly with respect to impaired OHRQoL.
- From an OHRQoL perspective subjects seem to prefer natural teeth above artificial teeth.
